# The Feasibility of Using Near-Infrared Spectroscopy and Chemometrics for Untargeted Detection of Protein Adulteration in Yogurt: Removing Unwanted Variations in Pure Yogurt

**DOI:** 10.1155/2013/201873

**Published:** 2013-06-16

**Authors:** Lu Xu, Si-Min Yan, Chen-Bo Cai, Zhen-Ji Wang, Xiao-Ping Yu

**Affiliations:** ^1^Zhejiang Provincial Key Laboratory of Biometrology and Inspection & Quarantine, College of Life Sciences, China Jiliang University, Xueyuan Street, Xiasha Higher Education District, Hangzhou 310018, China; ^2^Department of Chemistry and Life Science, Chuxiong Normal University, Luchengnan Road, Chuxiong 675000, China

## Abstract

Untargeted detection of protein adulteration in Chinese yogurt was performed using near-infrared (NIR) spectroscopy and chemometrics class modelling techniques. sixty yogurt samples were prepared with pure and fresh milk from local market, and 197 adulterated yogurt samples were prepared by blending the pure yogurt objects with different levels of edible gelatin, industrial gelatin, and soy protein powder, which have been frequently used for yogurt adulteration. A recently proposed one-class partial least squares (OCPLS) model was used to model the NIR spectra of pure yogurt objects and analyze those of future objects. To improve the raw spectra, orthogonal projection (OP) of raw spectra onto the spectrum of pure water and standard normal variate (SNV) transformation were used to remove unwanted spectral variations. The best model was obtained with OP preprocessing with sensitivity of 0.900 and specificity of 0.949. Moreover, adulterations of yogurt with 1% (w/w) edible gelatin, 2% (w/w) industrial gelatin, and 2% (w/w) soy protein powder can be safely detected by the proposed method. This study demonstrates the potential of combining NIR spectroscopy and OCPLS as an untargeted detection tool for protein adulteration in yogurt.

## 1. Introduction

Today, growing public concern regarding caloric and fat intake has raised the demand for low- or reduced-fat foods [1, 2]. Yogurt, a traditional dairy product produced by bacterial fermentation of milk with a starter culture containing *Streptococcus salivarius *ssp. *thermophilus *and *Lactobacillus delbrueckii *ssp. *Bulgaricus*, has been very popular for its reduced fat content, special texture, flavor, and tang, as well as its nutritional and health benefits beyond those of milk [3–5]. In China, the yogurt market constitutes a segment with great potential for expansion. In 2003, the consumption of yogurt and its related drinks was estimated to account for 11% of the total dairy production of China [6].

Although yogurt and its related products are favored by many people in China, various adulterations in milk and yogurt products have aroused great public concern about the quality and safety of yogurt. Since 2007, a series of scandals involving the adulteration and contamination of dairy products with melamine and other compounds such as cyanuric acid, ammeline, and ammelide have brought serious challenges to Chinese food quality supervision departments [7–12]. To deal with the crisis, numerous targeted analysis methods were developed to detect melamine and its analogues in dairy products, including liquid chromatography [13, 14], gas chromatography [15], laser Raman spectrometry [16], immunoassay [17], electrochemical analysis [18], flow injection analysis [19], and infrared spectroscopy [20–22] among others [23–25]. However, new reports on adulterations with illegal ingredients other than melamine and its analogues are popping up from time to time. A recent hot issue is the yogurt adulterations with nonmilk proteins, including vegetable protein powder, edible gelatin, and even industrial gelatin [26]. Obviously, if we just rely on targeted analysis methods, the adulterations would be out of control and the analysis would be trapped in a cycle of “adulteration, targeted analysis, and new adulterations,” and so on. Therefore, untargeted detection methods are required to enable the screening of dairy products for a range of known and unknown adulterants [27].

Untargeted detection of adulterations is a typical problem of chemometrics class modelling techniques [28, 29], which aim to answer the question of whether a new sample should be accepted or rejected by a sought-for class (e.g., pure and authentic yogurt). A class model proceeds as follows: (1) representative objects of the target class are collected and their characteristic signals are measured, (2) a class model is trained based on the measured signals to describe the distribution of authentic objects, and (3) a new object is analyzed and predicted by the class model. Some important issues should be considered when developing a class model. Firstly, to ensure the sensitivity of the class model, a training set of the representative authentic objects should be collected to include most if not all of the important variations of authentic products [30]. Second, the specificity of the class model should be validated by predicting future objects. 

As a rapid analysis method, near-infrared (NIR) spectroscopy has been widely used in food quality control for some advantages over traditional chemical analysis methods [31–34]: (1) no or limited sample preparation, (2) less analysis time and cost, (3) the potential for nondestructive and online analysis, and (4) the ability to simultaneously characterize multiple chemical components. Therefore, the objective of this paper was to develop a rapid method for protein adulteration identification of Chinese yogurt by diffuse NIR spectroscopy and chemometrics class modelling techniques. A recently proposed one-class partial least squares model [35, 36] was used to model authentic yogurt samples.

## 2. Materials and Methods

### 2.1. Pure and Adulterated Yogurt Samples

Fresh milk samples were collected from local dairies and were heated to about 80°C to kill any undesirable bacteria. The milk was then cooled to 40°C and a starter bacteria culture was added, and the temperature was maintained for 12 hours to allow fermentation in a fermentor. The prescribed starter culture (Hecan, Changzhou, China) includes *Streptococcus salivarius *ssp. *thermophilus *and *Lactobacillus delbrueckii *ssp. *Bulgaricus.* The fermentation was terminated by keeping the yogurt samples at −4°C and adulterating, and NIR measurements were performed in 12 hours. Adulterated yogurt samples were prepared by blending the previous pure samples randomly with different levels of adulterant solutions, including edible gelatin (Pucheng, Hangzhou, China), industrial gelatin (Hengtong, Foshan, China), and soy protein powder (Jichuan, Hangzhou, China). The thicknesses of pure and adulterated yogurt objects were kept to be approximately equal by adding pure water, which is the common practice of protein adulteration. The information concerning the 60 pure and 197 adulterated yogurt objects is listed in Table 1.

### 2.2. Acquisition of NIR Spectra

The NIR diffuse reflectance spectra of pure and adulterated yogurt samples were measured in the spectral range from 4000 to 12000 cm^−1^ on a Bruker TENSOR37 FTIR spectrometer (Bruker Optics, Ettlingen, Germany) using OPUS software. All spectra were measured in a quartz cup with a PbS detector and an internal gold background as the reference. The depth of yogurt in the quartz cup was about 5 cm. The resolution was 8 cm^−1^ and the scanning interval was 3.857 cm^−1^. Therefore, each spectrum had 2074 individual data points for multivariate analysis. Sixty-four scans were performed for each object and more scans did not improve the signal quality significantly. The spectrum of pure water was obtained by averaging the five repeated measurements of water membranes on the internal gold background.

### 2.3. Data Preprocessing and Splitting

To ensure the specificity of class models to detect potential adulterants, unnecessary variations in the target-class samples should be removed or reduced. Because water has strong absorbance in NIR range, the water variations in milk and during production, transportation, storage, and processing of yogurt can cause great signal variations in pure yogurt samples, which would make a class model wrongly accept more adulterated yogurt objects. Therefore, in order to remove the influence of water variations, the spectra of both training and prediction objects were orthogonally projected (OP) onto the complement space of water spectrum as follows:

(1) $$\begin{array}{c} x_{\text{new}}=\left(I-ss^{+}\right){x_{\text{raw}}}^{}, \end{array}$$

where “+” denotes the pseudoinverse of a matrix, respectively. **x**_raw_ and **x**_new_ are the vectors of raw and preprocessed spectra of an object and **s** is the spectrum of pure water, respectively. Standard normal variate [37] was originally designed to reduce scattering effects in the spectra but was also proved to be effective in correcting the interference caused by variations and reducing spectral backgrounds. Therefore, SNV transformation was also used as a preprocessing option.

 Representative training and prediction sets are required to train and validate a class model; in this paper, the DUPLEX algorithm [38] was used to divide the analyzed objects into a training set and a test set. DUPLEX algorithm proceeds as follows: (1) the two samples with largest Euclidean distance were selected and put in the training set, (2) the two objects with largest distance among the remaining samples were put in the test set, and (3) steps (1) and (2) are repeated until one has obtained as many test objects as predefined. By alternatively selecting the furthest samples for the training set and test set, DUPLEX can obtain two data sets almost with equal distributions in the experimental space. 

### 2.4. Class Modelling Technique

OCPLS was used to develop a class model for pure yogurt samples. OCPLS balances the explained variances and compactness of the target class by projecting each training object onto the class average. With a training matrix *X*  (*n*  by  *p*) including *p* features of *n* objects from the target class, OCPLS works in the framework of partial least squares (PLS) regression as follows:

(2) $$\begin{array}{c} 1=Xb_{\text{PLS}}+e, \end{array}$$

where each element of the response vector 1 (*n* by 1) is 1, **b**_PLS_ (*p* by 1) is the PLS regression coefficient vector, and **e**  (*n*  by  1) denotes regression errors. It should be noted that the training data **X** should not be column centered; otherwise, all the predictors would be orthogonal to the response vector 1. 

The model errors **e** can be assumed to have a normal distribution and its standard deviation can be seen as a measure of the target-class dispersion. The standard deviation of **e** can be estimated as the prediction errors of Monte Carlo cross-validation (MCCV) [39]. Given a significance level, *α*, the 1-*α* confidence interval of the predicted response value (*y*_*un*_) for accepting a new sample can be estimated as

(3) $$\begin{array}{c} \left(1-{\hat{\mu }}_{e}-z_{1-\alpha /2}\cdot \hat{\sigma },1-{\hat{\mu }}_{e}+z_{1-\alpha /2}\cdot \hat{\sigma }\right), \end{array}$$

where *z*_1−*α*/2_ is the critical value of standard normal distribution, ${\hat{\mu }}_{e}$ is the mean of **e**, and $\hat{\sigma }$ is the standard deviation of model error estimated by MCCV. 

To evaluate the performance of class models, sensitivity (Sens) and specificity (Spec) of prediction were used as follows:

(4) $$\begin{array}{c} \text{Sens}=\frac{\text{TP}}{\text{TP}+\text{FN}},\\ \text{ Spec }=\frac{\text{TN}}{\text{TN}+\text{FP}}, \end{array}$$

where TP, FN, TN, and FP denote the numbers of true positives, false negatives, true negatives, and false positives, respectively. Pure and adulterated yogurt objects were denoted as positives and negatives, respectively.

## 3. Results and Discussion 

**Figure 1:**
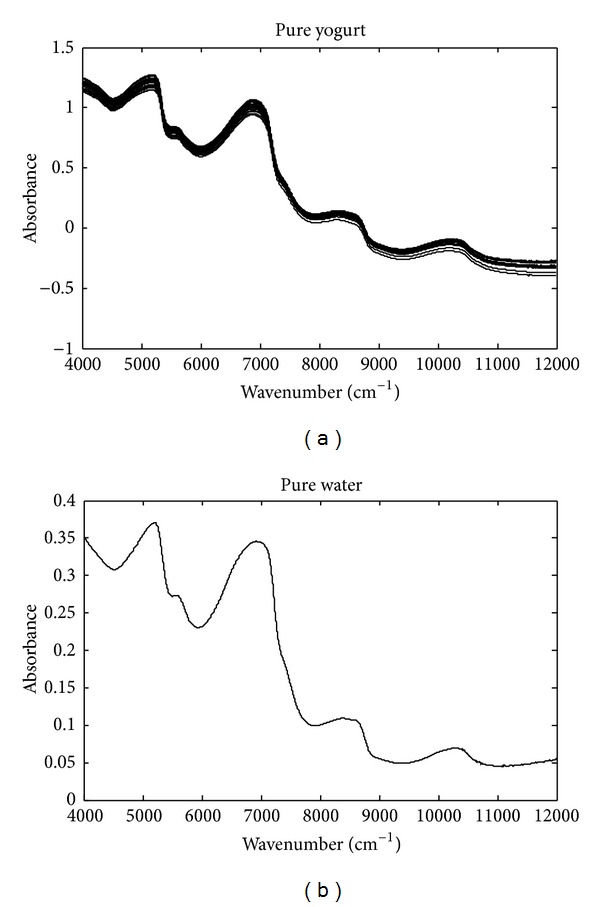
Typical raw NIR spectra of pure yogurt objects and the spectrum of pure water.

**Figure 2:**
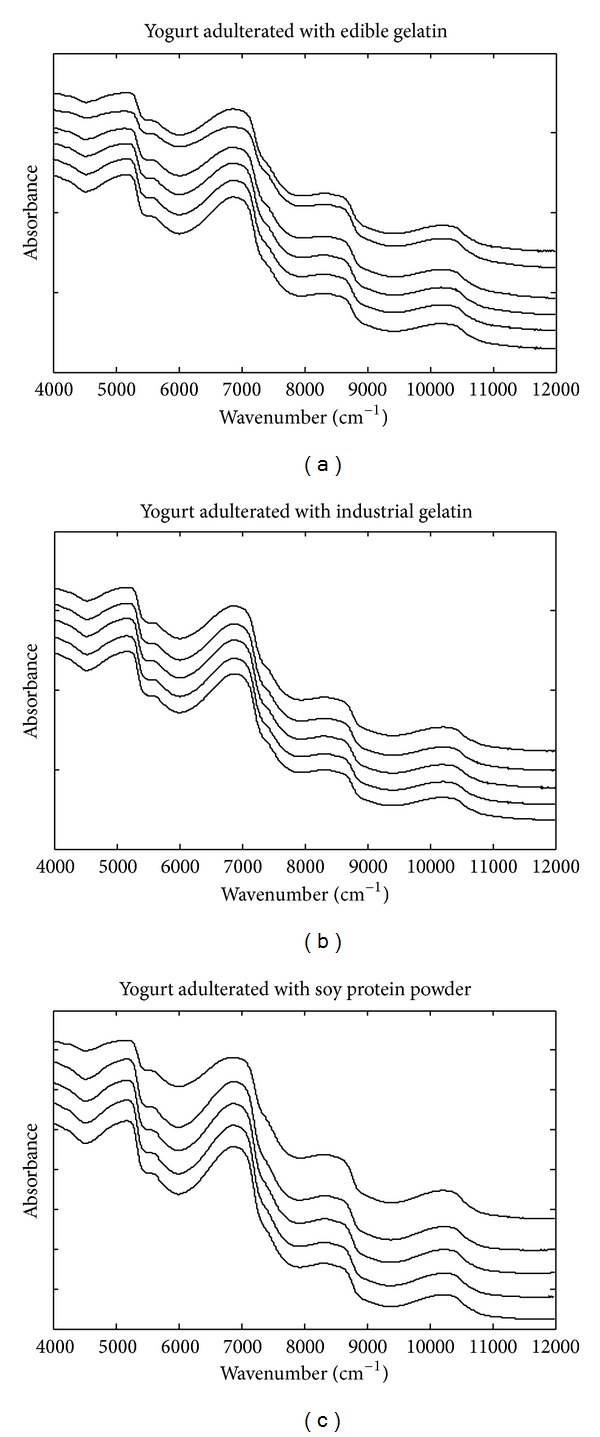
Average NIR spectra of yogurt objects adulterated with different levels of edible gelatin, industrial gelatin, and soy protein powder. A shift was added to differentiate doping levels and a larger shift corresponds to a higher doping level.

The raw NIR spectra of pure yogurt and water are shown in Figure 1.  Figure 2 demonstrates the raw spectra of yogurt objects adulterated with different levels of edible gelatin, industrial gelatin, and soy protein powder. In each subplot of Figure 2, a shift was added to differentiate doping levels. As demonstrated in Figures 1 and 2, the spectra of pure water and pure and adulterated yogurt samples have almost same absorbance bands in the range of 4000–12000 cm^−1^, which can be attributed to the high contents and the strong absorbance of water. Because of peak overlapping, the raw spectra have a very poor resolution, and the spectra variations caused by other chemical components were seriously masked by the absorbance bands of water. Therefore, proper spectra preprocessing is required to remove the unnecessary spectral variations due to water and highlight the spectral variations caused by adulterants.

**Figure 3:**
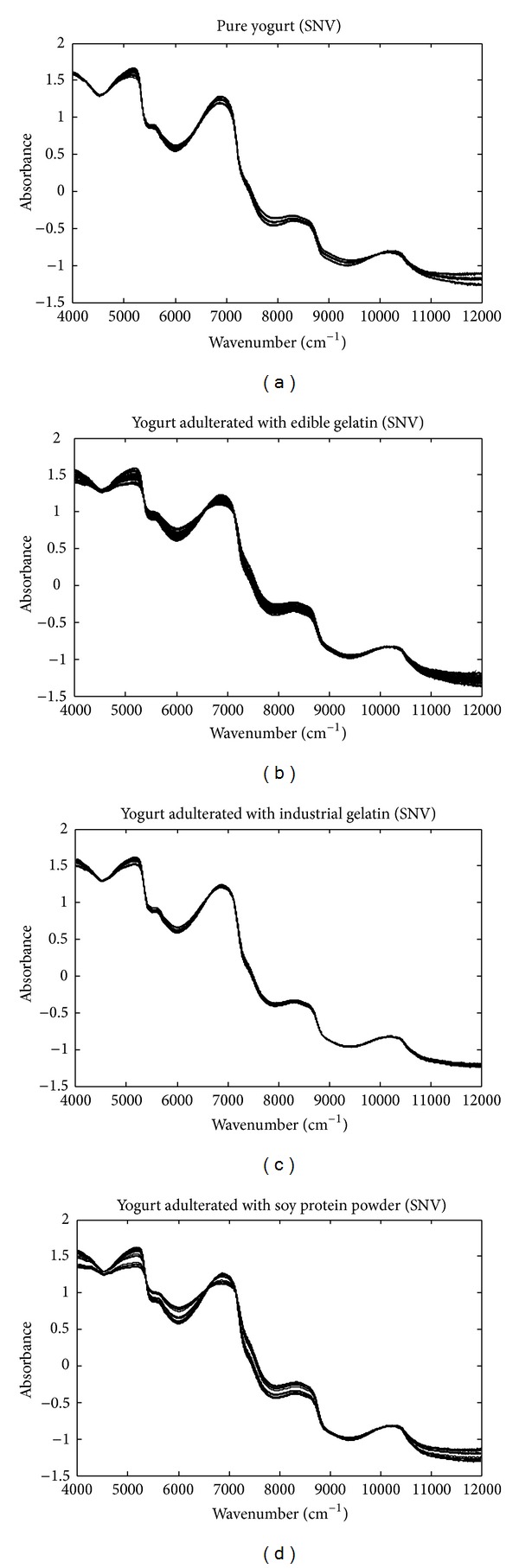
Typical SNV-transformed NIR spectra of yogurt objects adulterated with different levels of edible gelatin, industrial gelatin, and soy protein powder.

**Figure 4:**
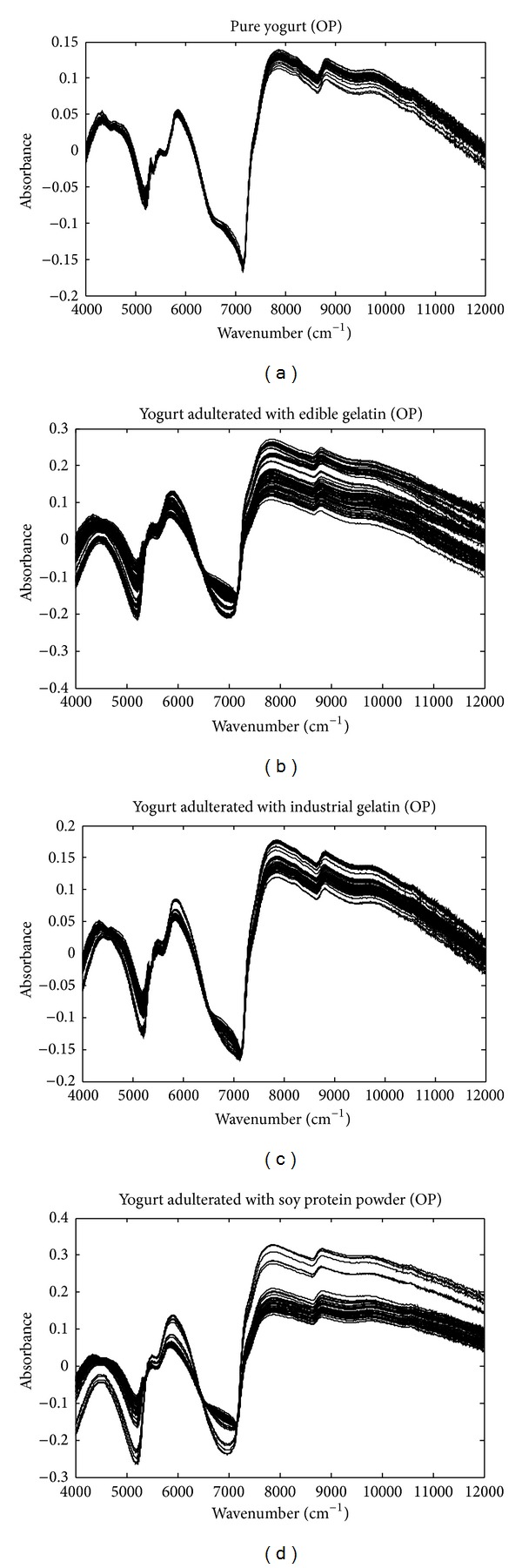
Typical orthogonally projected (OP) NIR spectra of yogurt objects adulterated with different levels of edible gelatin, industrial gelatin, and soy protein powder. All the spectra were projected onto the orthogonal complement space of water spectrum.

SNV-transformed spectra were shown in Figure 3. SNV can reduce some spectral variations while enhancing others. All the raw spectra were also projected onto the orthogonal complement space of water spectrum.  Figure 4 demonstrates the OP spectra of pure and adulterated yogurt objects. Then, the DUPLEX algorithm was used to split the 60 pure yogurt samples into a training set of 40 samples and a test set of 20 samples. All the 197 adulterated yogurt samples were used as “negative” test samples to test the specificity of the class model. Therefore, the training set had 40 “positive” (pure) yogurt objects and the test set included 20 “positive” and 197 “negative” (adulterated) objects. Based on the raw, SNV-transformed, and OP spectra, OCPLS models were developed and the model performance was evaluated by sensitivity and specificity obtained from the prediction of the test objects. MCCV with 10% left-out samples was used to determine the number of latent variables and the repeat number was 100. The predicted residual sum of squares (PRESS) values by MCCV were examined and the number of components was determined to obtain a low PRESS value, and more components cannot reduce the PRESS value significantly. 

**Figure 5:**
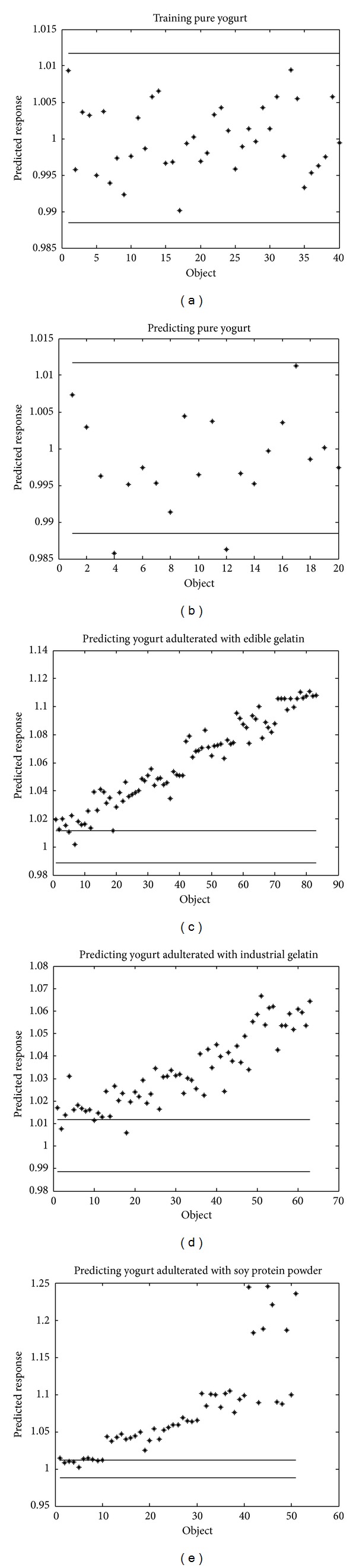
Training and predicting results obtained by orthogonally projected (OP) spectra and 5-component OCPLS model. For adulterated objects, the levels of adulterants, edible gelatin (1%, 2%, 3%, 4%, 6%, and 8%), industrial gelatin (0.5%, 1%, 2%, 3%, and 5%), and soy protein powder (0.5%, 1%, 2%, 3%, and 5%) are arranged in an ascending order along the *x*-axis (object).

The significance level of OCPLS was set to be 0.05. The training and prediction results of OCPLS models were summarized in Table 2. As seen from Table 2, both SNV and OP spectra preprocessing can improve the specificity of class models by reducing the unwanted variations caused by water absorbance and backgrounds. The best model was based on OP spectra with sensitivity of 0.900 and specificity of 0.949, respectively. The prediction results obtained with OP spectra were demonstrated in Figure 5. In Figure 5, the adulterated yogurt samples were arranged according to an ascending doping level. With both OP- and SNV-preprocessed spectra, the wrongly accepted samples (false positives) had a doping concentration of 0.5% for edible gelatin, 1% for industrial gelatin, and 1% for soy protein powder, respectively. Moreover, the distance of an adulterated object to the critical value increases with doping level, indicating that the identification of higher doping levels, namely, 1% edible gelatin, 2% industrial gelatin, and 2% soy protein powder, can be safely detected.

## 4. Conclusions 

NIR spectroscopy combined with a recently proposed chemometrics class modelling method, OCPLS, has shown much potential for developing “untargeted” detection for protein adulteration in Chinese yogurt samples. In the current experimental conditions, adulterations with edible gelatin (1%), industrial gelatin (2%), and soy protein powder (2%) can be detected by OCPLS with proper preprocessing of spectra. The results demonstrate that orthogonally projection of raw spectra onto the spectra of pure water is useful to remove unwanted variations. If the within-class variations are controlled effectively, the model specificity can be improved and lower levels of adulterants can be detected. 

## Figures and Tables

**Table 1:** Pure and adulterated yogurt samples analyzed.

No.	Adulterant^a^	Doping level (w/w)	Sample size
1	A0	0	17
2	A0	0	25
3	A0	0	18
4	A1	1%	12
5	A1	2%	14
6	A1	3%	14
7	A1	4%	15
8	A1	6%	15
9	A1	8%	13
10	A2	0.5%	14
11	A2	1%	9
12	A2	2%	12
13	A2	3%	13
14	A2	5%	15
15	A3	0.5%	10
16	A3	1%	10
17	A3	2%	10
18	A3	3%	10
19	A3	5%	11

^a^A0: pure yogurt; A1: edible gelatin; A2: industrial gelatin; A3: soy protein powder.

**Table 2:** Predicting results of OCPLS models for pure and adulterated yogurt objects.

Preprocessing	LVs^a^	Pure yogurt	A1	A2	A3	Sensitivity^b^	Specificity^c^
Raw spectra (*μ* = 0.0998, *σ* = 0.00644)^d^	7	3^e^	12	6	16	0.850 (17/20)	0.827 (163/197)
SNV (*μ* = 1.0004, *σ* = 0.00621)	5	2	3	5	8	0.900 (18/20)	0.919 (181/197)
OP (*μ* = 1.0001, *σ* = 0.00592)	5	2	2	3	5	0.900 (18/20)	0.949 (187/197)

^a^The number of PLS components.

^b^The numbers in the brackets denote TP/(TP + FN).

^c^The numbers in the brackets denote TN/(TN + FP).

^d^Model parameters.

^e^The number of objects that were wrongly predicted.
